# Ultrafast ultrasound imaging pattern analysis reveals distinctive dynamic brain states and potent sub-network alterations in arthritic animals

**DOI:** 10.1038/s41598-020-66967-x

**Published:** 2020-06-26

**Authors:** Line Rahal, Miguel Thibaut, Isabelle Rivals, Julien Claron, Zsolt Lenkei, Jacobo D. Sitt, Mickael Tanter, Sophie Pezet

**Affiliations:** 1 Laboratory of Brain Plasticity, ESPCI Paris, PSL Research University, CNRS UMR 8249, 10 rue Vauquelin, 75005 Paris, France; 2 Physics for Medicine Paris, Inserm, ESPCI Paris, CNRS, PSL Research University, Paris, France; 3 Equipe de Statistique Appliquée, ESPCI Paris, PSL Research University, UMRS 1158, 10 rue Vauquelin, 75005 Paris, France; 4 Center of Psychiatry and Neurosciences, INSERM U894, 102 rue de la Santé, 75014 Paris, France; 5 Institut du Cerveau et de la Moelle, INSERM U1127, CNRS UMR 7225, Sorbonne University, UPMC Univ Paris 06 UMR, S 1127 Paris, France

**Keywords:** Chronic pain, Prognostic markers

## Abstract

Chronic pain pathologies, which are due to maladaptive changes in the peripheral and/or central nervous systems, are debilitating diseases that affect 20% of the European adult population. A better understanding of the mechanisms underlying this pathogenesis would facilitate the identification of novel therapeutic targets. Functional connectivity (FC) extracted from coherent low-frequency hemodynamic fluctuations among cerebral networks has recently brought light on a powerful approach to study large scale brain networks and their disruptions in neurological/psychiatric disorders. Analysis of FC is classically performed on averaged signals over time, but recently, the analysis of the dynamics of FC has also provided new promising information. Keeping in mind the limitations of animal models of persistent pain but also the powerful tool they represent to improve our understanding of the neurobiological basis of chronic pain pathogenicity, this study aimed at defining the alterations in functional connectivity, in a clinically relevant animal model of sustained inflammatory pain (Adjuvant-induced Arthritis) in rats by using functional ultrasound imaging, a neuroimaging technique with a unique spatiotemporal resolution (100 μm and 2 ms) and sensitivity. Our results show profound alterations of FC in arthritic animals, such as a subpart of the somatomotor (SM) network, occurring several weeks after the beginning of the disease. Also, we demonstrate for the first time that dynamic functional connectivity assessed by ultrasound can provide quantitative and robust information on the dynamic pattern that we define as brain states. While the main state consists of an overall synchrony of hemodynamic fluctuations in the SM network, arthritic animal spend statistically more time in two other states, where the fluctuations of the primary sensory cortex of the inflamed hind paws show asynchrony with the rest of the SM network. Finally, correlating FC changes with pain behavior in individual animals suggest links between FC alterations and either the cognitive or the emotional aspects of pain. Our study introduces fUS as a new translational tool for the enhanced understanding of the dynamic pain connectome and brain plasticity in a major preclinical model of chronic pain.

## INTRODUCTION

Neuroimaging has proven to be an invaluable tool for the study and fundamental understanding of a wide range of neurological pathologies. Over the last two decades, resting state functional connectivity has shed light on the spatiotemporal organization of spontaneous activity of brain networks in neurological/psychiatric disorders and related animal models^1,2^. Infra-slow oscillations have been described, through different imaging modalities, such as blood oxygenation level^3^ arterial spin labelling perfusion fMRI^4^, MEG^5^ and fUS imaging^6^. (See for review^7,8^). Classically analyzed using averaged signals in selected regions of interest over the imaging time (6–10 min), a new insight into the fundamental nature of FC is to recognize the fluctuating nature of resting state signals over time. As a consequence, analysis of the dynamic functional connectivity (dFC) has recently been the focus of intense work from several teams, providing important insights on the brain functions, such as the neurobiological basis of consciousness^9–11^ and neuropathological mechanisms of schizophrenia^12^ and autism^13^. As reviewed by Pretri *et al*.^8^, several approaches in this analysis are currently being used: either using sliding windows, dynamic graph analysis or extracting dFC states using several mathematical approaches^8^.

Chronic pain pathologies, which are due to maladaptive changes in the peripheral and/or central nervous systems, are debilitating diseases that affect 20% of the European adult population ^14^. While preclinical studies are focusing on defining molecular mechanisms underlying these persistent pain states in animal models of human pathologies, neuro-imaging studies in human are mainly focusing on the identification of brain areas activated by painful stimuli and their alterations in chronic pain states.

Clinical studies investigating changes of FC in chronic pain patients’ studies revealed specific alterations of connectivity patterns in major hubs of the “pain matrix”, such as a reduced anti-correlation between the Default-mode network and the salience network^15^ and an increased connectivity between the insula and the anterior cingulate cortex^16^. However, the results obtained by these studies are sometimes inconclusive and/or contradictory, because chronic pain disorders represent numerous and diverse syndromes, each one having its own etiology. Also, clinical FC suffers the high inter-subject variability due to genotypic variations and the personal history of each patient. Despite the drawbacks associated with the use of animal models of human pathologies, preclinical studies have the advantage of using animals that are genetically similar to each other and also allow molecular, pharmacological and behavioral studies, that are key approaches for the development of new therapeutic strategies.

Our study aimed at studying alterations of brain FC and brain states in a clinically-relevant animal model of chronic inflammatory pain, Adjuvant-Induced Arthritis (AIA), by using a new ultrasound-based neuroimaging technique, functional ultrasound (fUS) imaging. By exploiting the hemodynamic impulse response to neural events^17^, this technique allows imaging of cerebral blood volume (CBV)^18,19^ in the rat^20^ and infant brain^21^ with a large field of view and high spatial (10–100 μm) and temporal (2 msec) resolution^20^, allowing detection in single trials and at the level of a voxel^22^. Due to neurovascular coupling, fUS imaging measures with high sensitivity both the cortical hemodynamic changes induced by olfactory^23^, visual^24^ and auditory^25^ stimuli. We also showed that this technique can image functional connectivity^6^ in a very reproducible manner in anesthetized animals.

Aiming to overcome the technical difficulties to image resting state networks and analyse FC in animal models using classical neuroimaging techniques^26^, our study aimed to investigate in depth the alterations of FC and dFC, by using fUS imaging. Our results provide a novel approach for the study of dynamic alterations of brain networks in inflammatory chronic pain condition.

## Materials and Methods

### Animals

All experiments were performed in agreement with the European Community Council Directive of 22^nd^ September (010/63/UE) and the local ethics committee (“Comité d’éthique en matière d’expérimentation animale n°59, ‘Paris Centre et Sud’”, project agreement APAFIS#13363-2018020321116321-V3). Experiments were performed in accordance with these guidelines and regulations previously quoted, and within the strict project agreed by the ethic committee and the French ministry of Research. Experiments required 26 male OFA Sprague Dawley rats (Charles River, Saint-Germain-Nuelles, France) weighing 225–250 g at the beginning of the experiment. Animals arrived in the laboratory two weeks before the experiment. They were kept two per cage at a constant temperature of 22 °C, with a 12-hours alternating light/dark cycle (light 8 AM – 8 PM). Food and water were available *ad libitum*.

### Experimental paradigm

The aim of this study was to image the changes of FC in arthritic animals. Because ultrasound waves are highly attenuated by the rat skull, the skull needs to be removed completely, which is a very invasive procedure used either in terminal studies, or in chronic studies, the skull can be replaced by fUS compatible material. In a preliminary study, we tried to perform a longitudinal study using chronically implanted TPX plastic to replace the skull, as performed by other teams^27,28^. However, due to the constraint of our study, i.e. the large size of the window necessary (1 cm × 2 cm) and the time laps of our experiment (seven weeks), the quality of the window was too variable and we turned to an experimental design that would include two animal groups (control and arthritic), imaged four weeks after the model induction, using in all animals a thinned-skull window prepared four days before the imaging session. This procedure, that cannot be used for longer than a week because the bone grows back again, was previously used by us and others^6,29,30^. It has the advantage of keeping the brain’s integrity, avoiding any inflammation and leaving the animal to recover before the imaging session, hence providing strong reproducibility in the results obtained^6^.

**Figure 1:**
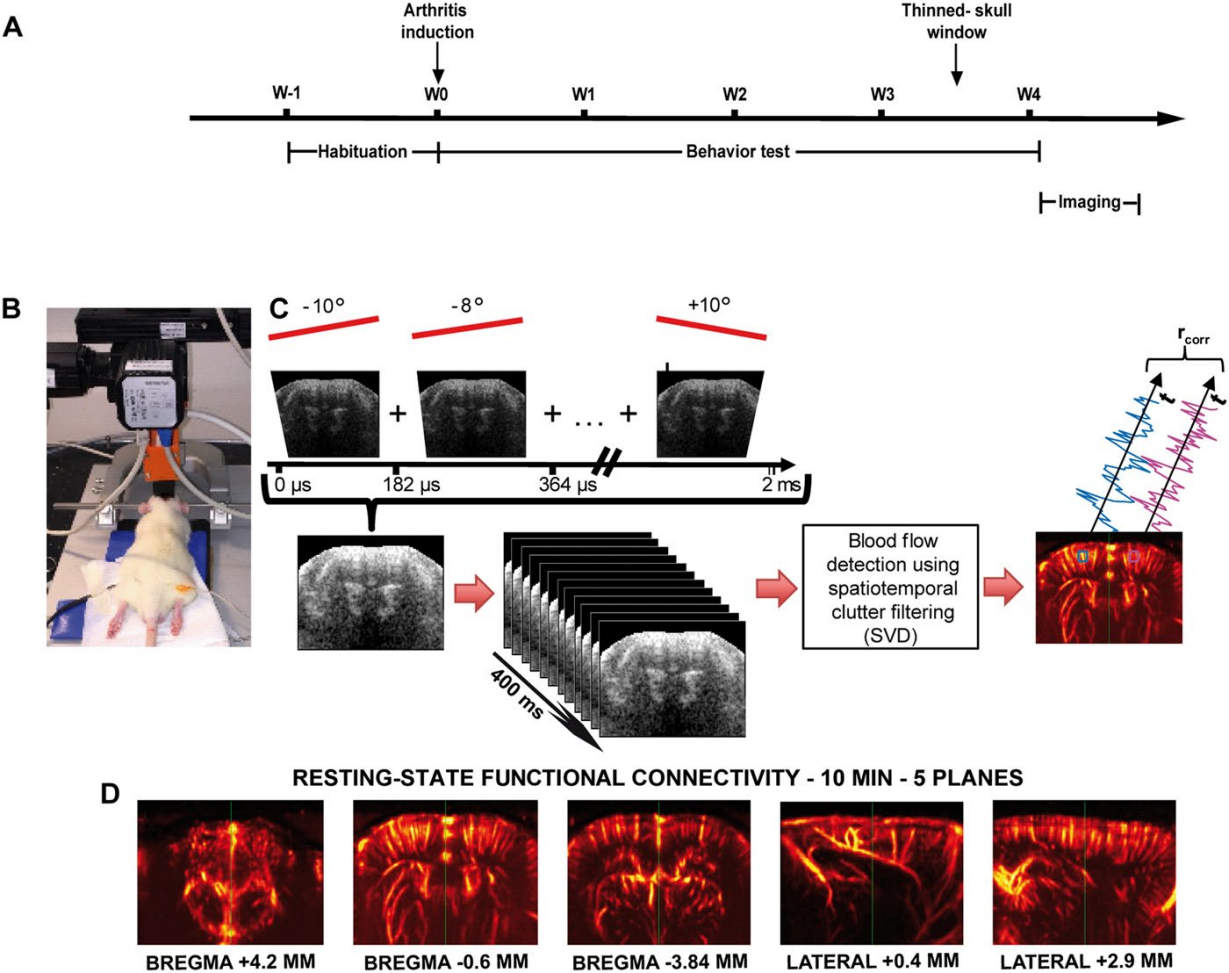
Experimental design. (**A**) Time line of the experimental design. Behaviour habituation and tests were performed during the development of the model. A thinned-skull window was made four days before the imaging session. (**B**) Setup of the fUS imaging experimentation. The rat was placed on a stereotaxic frame over a warm plate and is continuously perfused with the anesthetic mixture ketamine/medetomidine via a syringe pump. Its arterial pressure, as well as heart and respiratory rates were measured periodically. The ultrasound probe was positioned above the previously thinned skull, with echographic gel in between. The probe was moved using the 4-axis motor. (**C**) 11 tilted planar ultrasonic waves were emitted into the rat brain. The coherent sum of the resulting 11 images produced a compound image every 2 ms. The final Doppler image was the mean image over a 400 ms acquisition period, i.e., 200 compound images. The CBV signal was then extracted using a “clutter” filter that removed the tissue signal, leaving the blood signal intact. Computing the signal of each pixel in the plane over ten minutes, we correlated them two-by-two to study FC (**D**) Vascular signal of the planes imaged during a 10-minute resting-state acquisition session: 3 coronal planes and 2 sagittal planes.

Clinical and behavioral tests were performed before induction of the model and once per week for three weeks for the AIA group. The thinned-skull window was prepared four days before the imaging session (see below, Fig. 1A). The animals were killed at the end of the imaging session.

### Induction of long-term inflammatory pain

Adjuvant-Induced Arthritis (AIA) is a well characterized^31,32^ and clinically-relevant model of polyarthritis induced in male OFA Sprague Dawley rats (Charles River, Saint-Germain-Nuelles, France) by a bilateral injection (50 µl in each hind paw under isoflurane anesthesia) of a mixture of 6 mg of Mycobacterium butyricum (Difco Laboratory, USA) suspended in 1 mL of an emulsion of liquid paraffin/0.9% NaCl/Tween80 (6:4:1 v/v/v). In parallel, control animals received 50 µL of saline. This study required two groups of arthritic rats (N = 9 for the group n°1, required for the main part of the study and N = 8 for the group n°2, required for the validation of the ROC analysis) and one group of control rats (N = 9).

### Clinical and behavioral studies

Habituation: The animals were subjected to a two-week habituation period before the beginning of the experiment. During this period, animals were habituated to the room, the experimenter, and increasingly to the different tests (they were habituated to being handled and kept without being restrained into a box and to the electronic von Frey testing).

To assess the evolution of polyarthritis, the four following parameters were measured once a week (The statistical analysis of all these tests was performed using the Mann-Whitney U test):

1. Weight gain (difference in individual weight between weeks 3 and 0).
2. The scoring of peripheral inflammation was performed as described by Bas *et al*.^33^. A one-point score was given for each inflamed toe/knuckle, five points for an inflamed paw and five points for an inflamed wrist or ankle^33^. This results in a cumulative score ranging from 0 to 15 for each paw, giving a global score out of 60.
3. The static mechanical allodynia was measured as previously described^34^. Briefly, rats were individually placed on an elevated wire mesh floor in a clear plastic cylinder (22 cm in diameter) and were adapted to the testing environment for 10 min. An electronic von Frey hair unit (EVF-3; Bioseb, France) was applied to the midplantar area of each hind paw. Paw sensitivity threshold was defined as the minimum pressure eliciting a robust and immediate withdrawal reflex of the paw. The stimulus was applied on each hind paw 3 times with a 5-s interval, and the value adopted as a threshold for a rat was the average of the values measured. Mechanical allodynia was defined as a significant decrease in withdrawal thresholds to EVF-3 application.
4. The allodynic state of the ankle to articular movement was tested, while the animal was calmly sitting, using the “foot-bend” test. This consisted in the bending of each hind paw, which may elicit a vocalization (score of 1)^31^. This measure was repeated five times on each hind paw, with a score ranging from 0 to 5 for each paw.

### Preparation of a bilateral thinned-skull window

In order to avoid attenuation of the ultrasonic waves, the skull was thinned four days before each imaging session using a previously described procedure^6,29^. Briefly, animals were anesthetized using an initial intraperitoneal injection of a mix of medetomidine (Domitor®, 0.3 mg/kg) and ketamine (Imalgène®, 40 mg/kg), which was extended one hour later by a subcutaneous injection of the same mix of anesthetics at a dose of 10 mg/kg/h Imalgène® and 0.07 mg/kg/h Domitor®, as previously described^6^.

While the head of the animal was held in a stereotaxic frame, a cranial window of thinned skull measuring 1.5 cm long x 1 cm wide was prepared starting 5 mm anterior of the Bregma to the Lambda^35^. During the thinning procedure, three layers of skull bone were consecutively removed by drilling (Foredom, USA) at low speed, using a micro drill steel burr (Fine Science Tools, cat. no. 19007-07) with frequent cooling, as described in Yang *et al*.^29^. Finally, the skin was sutured using 5.0 non-absorbable Ethicon thread. The anesthesia was then reversed with a subcutaneous injection of atipamezole (Antisedan®, 1 mg/kg). Post-surgical pain was prevented by subcutaneous injection of meloxicam (Metacam®, 0.2 mg/kg).

### Choice of imaging planes

Functional ultrasound (fUS) imaging enables the measurement of the CBV with high sensitivity and high spatiotemporal resolutions in the rat and mice brain^20,23,24,36,37^. However, compared to fMRI, fUS imaging suffers so far a lack of simultaneous imaging in the three dimensions of space. As a consequence, imaging in this study was performed in 2 dimensions planes including the regions of interest to be studied. Based on previous description of FC alterations in chronic pain patients^4,38–41^ and animal models^42^, we hypothesized that in arthritic animals, the default mode network, the reward circuit and the sensory-motor network might undergo significant functional alterations and that these alterations could provide interesting insights and quantification for animal’s classification. This is particularly interesting in pre-clinical research for the evaluation of drug’s efficacy. In order to test our hypothesis, we chose the 3 following coronal planes: Bregma +4.2 mm, Bregma −0.6 mm, Bregma −3.84 mm, and the 2 following sagittal planes: lateral +0.4 mm and lateral +2.9 mm (Fig. 1D). The sagittal planes aimed for the structures of the reward and the default mode networks.

### fUS imaging

For imaging sessions, animals were anesthetized using an initial intraperitoneal injection of medetomidine (Domitor®, 0.3 mg/kg) and ketamine (Imalgène®, 40 mg/kg), followed by a maintenance dose of medetomidine (0.1 mg/kg/h) and ketamine (12.5 mg/kg/h) using a syringe pump. The body temperature of the rats was kept constant using a heating blanket (Harvard Apparatus, Holliston, MA, USA). The physiological parameters of the animal were followed (heart and breathing rates) using MouseOxplus (STARR Life Science, Oakmont, USA). The skull was gently cleaned with saline solution and covered with previously centrifuged echographic gel. Acquisitions were performed when the animals had reached a physiological temperature and showed reproducible FC matrices (i.e. approximately 45 min-1h after induction, as previously observed^6^). Two scans were performed, one in the antero-posterior and another in the sagittal direction (Fig. 1D), using a 4-axis motor on which the ultrasound probe was fixed (Fig. 1B). This setup allowed us to target the five brain planes of interest for the rest of the experiment (see above).

### fUS acquisition sequence

fUS imaging was performed using a linear ultrasound probe (128 elements, 15 MHz central frequency, 100 μm spatial pitch and 8 mm elevation focus, Vermon, Tours, France) driven by an in-house ultrafast ultrasound scanner (Inserm Accelerator of Technological Research in Biomedical Ultrasound, Paris, France). The ultrasound sequence operated as follows: the rat brain was insonified by a train of ultrasonic tilted plane waves^43^ with angles varying from −10° to +10° with a 2° step (Fig. 1C), with a Pulse Repetition Frequency of 5.5 kHz. The backscattered echoes were recorded by the transducer array and beamformed to produce a high-quality image, which is the coherent sum of the 11 tilted plane waves. This sequence was repeated 200 times at a 500 Hz sampling rate. These 200 raw ultrasonic images produced a single ultrasensitive Doppler image every 400 ms. Biological nuisances were removed as follows: to remove the tissue signal and extract the CBV signal from raw data, we used a spatio-temporal clutter filter based on the singular value decomposition (SVD) algorithm. By exploiting the spatiotemporal coherence of backscattered signals^44^, this SVD filter was previously validated for its high efficiency to separate tissue and blood motion (strongly outperforming all conventional tissue/flow motion filters). It allows such a filtering, while enabling the detection of microvascular flow. Despite its clear benefits, it cannot be excluded that some residual noise could remain in the final time series and affect the estimates of the connectivity matrix similarly for all groups. Finally, a power Doppler image was obtained by the incoherent averaging of the CBV in each pixel every 400 ms. We showed previously^6^ that averaging the ultrafast Doppler signal samples acquired at 0.5 kHz over 400 ms enables a good measurement of FC in anaesthetized rats and a complete suppression of cardiac pulsations and breathing artifacts from the resting state bandwidth. Moreover, as already shown in Demene *et al*.^44,45^ most of the energy contained in the raw ultrasonic Doppler signal comes from a Doppler frequency bandwidth ranging between 40 Hz and 150 Hz which corresponds to typical blow flow speeds ranging between 2 mm/s and 7 mm/s. Such blood flow speeds in brain vessels correspond to typical arteriole and venule diameters ranging between 5 µm and 50 µm as summarized in a meta-analysis of vascular physiology in different animal models by Piechnik *et al*.^46^. To optimize the computation time of the ultrafast scanner data processing software, a “mask” delineating the pixels of interest was drawn such as to not beamform the signal of the skull or the echographic gel. The brain was continuously insonified over a ten-minute period, resulting in a 3D Doppler array [n_z_ = 78, n_x_ = 128, n_t_ = 1500].

Five different planes were imaged: three coronal planes at Bregma +4.2 mm, Bregma −0.6 mm and Bregma −3.84 mm and two sagittal planes at Lateral +0.4 mm and Lateral +2.9 mm (Fig. 1D). These planes are known to contain several regions involved in pain signal processing. We were then able to plot the CBV time course over the ten-minute acquisition for each pixel of the imaged planes, as shown in Fig. 1C.

### Seed-based analysis

For each ten-minute acquisition, the Doppler signal was filtered in the resting-state frequency band [0.05–0.2] Hz, as in previous studies^6^, and normalized by the square root of its energy. A correlation map was constructed by computing Pearson’s correlation coefficient r_ROI1,pix i_ between the mean temporal Doppler signal in a selected, often large, seed Region Of Interest (ROI) chosen as the reference signal and the signal of each pixel i of the image. This revealed global changes in connectivity between distinct ROIs in different planes of the brain. Then, in each imaged plane, the mean normalized Doppler signal was computed for two selected ROIs, and Pearson’s correlation coefficient r_ROI1,ROI2_ between the two signals was computed. To compare these correlation coefficients between control and arthritic rats, they were Fisher-transformed to normalize their distributions. In cases of normality, Welch’s test was performed; otherwise, the non-parametric Mann-Whitney test was used. Benjamini-Hochberg’s correction for multiple comparisons was performed for each plane dataset, and a false discovery rate of 0.05 was adopted. Seed ROI regions were chosen based on a hypothesis-driven approach.

### Correlation matrix analysis

For each acquisition, a correlation matrix was constructed as previously described^6^, based on the rat Paxinos atlas^35^ at Bregma −0.6 mm and using the filtered and normalized signals of the seed-based analysis. Correlation matrices were constructed for all rats of the control group and all rats of the arthritic group. Once again, the correlation coefficients between the signals of each couple of ROIs were Fisher-transformed; if the transformed data were normally distributed, a parametric Welch test was performed; otherwise, the Mann-Whitney test was applied. We performed Benjamini-Hochberg’s correction for multiple comparisons. The ‘significance matrix’ shows the pairs of ROIs that have a significantly different correlation coefficient between the arthritic and control groups, with a false discovery rate of 0.05.

### Dynamical FC, unsupervised clustering (k-means algorithm)

#### Phase matrices computation

Phase-based dynamic FC was preferred over a sliding-window approach because the former approach increases the temporal resolution by avoiding the inclusion of overlapping signals. As the Doppler spectrum of the fUS signal was exhibiting a power-law trend, the dominant carrier-frequency corresponds to the lowest frequency of the Doppler spectrum (0.05 Hz). The Hilbert transform was used to calculate inter-ROI phase differences for each image. This approach treats the ROI signal f(t) as the real part of a complex signal z(t), with an imaginary component as the Hilbert transform of f(t): z (t) = f(t) + iH[f(t)]. The phase was calculated as the inverse tangent of the ratio of the imaginary and real signals^47^.

For each animal, we computed matrices containing the cosines of the phase difference over time of each pair of ROIs used in the correlation matrix analysis. To compute the phase signal in an ROI, its mean Doppler signal was calculated and normalized. This is then filtered in the resting-state band of [0.05–0.2] Hz^6^ and Hilbert-transformed as described previously^47^. The phase signal φ(t) was then extracted for each ROI. Next, the phase signals were truncated of their first and last hundred samples, reducing their duration from 600 s to 520 s. A 3D phase-locked matrix Mpl was then constructed as: M_PL (i,j,t)=cos(φ_i (t)-φ_j (t)), where i and j vary from 1 to 10, with 10 as the total number of ROIs selected and t as time.

#### Unsupervised clustering and brain states extraction

We performed an unsupervised learning of the brain-states using the k-means clustering algorithm with the cityblock (L1) distance of the cosine of the phase difference, as previously described^9^. The number of clusters *k* was varied from 5 to 7, with robust results. To avoid local-minima during the clustering procedure we replicated 200 times the algorithm for each run and kept the replication that minimized the sum of within-cluster distances. For each number of clusters, we obtained a decomposition basis of brain states with diverse probabilities of occurrence throughout the ten minutes of resting-state fluctuations. The occurrence rate of each state for each individual was calculated as the total number of volumes for which that given state was present divided by the total number of volumes of the acquisition. To evaluate possible differences in the occurrence of some brain states according to the arthritic state of the rats, we compared the observed frequencies between the control and arthritic groups with Welch’s test in cases of Gaussian distributions and Mann-Whitney’s test in other situations, with a type I error risk of 0.05. Benjamini-Hochberg’s correction for multiple comparisons was applied and a false discovery rate of 0.05 was adopted.

### Correlation matrices of FC alterations and behavior

To investigate a possible link between the development of FC alterations and chronic pain behavior, we correlated FC alterations (as characterized by the seed-based, correlation matrix and FC dynamics) with behavior. With the seed-based analysis, we constructed a vector containing the correlation coefficients of the significantly altered ROI pairs and the behavioral data for each rat. As described previously^6^, we correlated each element of the vector with all the other elements. The resulting correlation matrix can be decomposed in three parts: first, the top left is the correlation of the seed-based dataset with itself; second, the top right is the correlation of the data with behavior; third and last, the bottom left is the correlation of the behavioral dataset with itself. We performed the same procedure for the correlation matrix analysis, with a vector containing the significantly altered correlations in addition to the behavioral data. Finally, for the k-means clustering analysis, the vector contained the occurrence rates of each of the seven brain states in addition to the behavioral data.

### ROC curves

Using the results of the seed-based analysis, we were able to plot sensitivity-specificity ROC curves using MedCalc Statistical Software version 16.4.3 (MedCalc Software bvba, Ostend, Belgium; https://www.medcalc.org; 2016). A ROC curve represents the true positive rate, or sensitivity, as a function of the false positive rate. In order to assess the corresponding diagnosis power, ROC curves were plotted using a logistic model output containing the correlation values of the seed-based analysis, with an increasing number of ROI pairs included in the calculation.

The first ROC curve enabled the determination of the correlation threshold, using the first cohort of arthritic rats (N = 9) and a subset (N = 5) of the control animals (“Test cohort”). A second cohort of independent animals, the “Validation cohort” consisting of (N = 8) arthritic rats and another subset of (N = 4) control animals was used to backcross the previous findings. Using the threshold defined above (using the “Test cohort”), the sensitivity and specificity were measured using seed-based data from animals of the “Validation cohort”.

### Data and code availability statement

All data supporting the findings of this study associated with figures are available. These data can be downloaded in the supplementary materials of the publication on the repository website ‘Zenodo’ under the name ‘Dataset Raw data of the article ‘Ultrafast ultrasound imaging pattern analysis reveals distinctive dynamic brain states and potent sub-network alterations in arthritic animals”, (DOI 10.5072/zenodo.520533).

## Results

### Behavior

**Figure 2:**
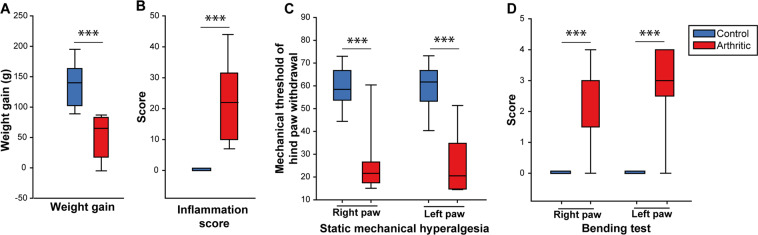
Arthritic rats display (3 weeks after the induction of the model) a sustained peripheral inflammation, reduced body weight gain and mechanical hypersensitivity. Clinical (**A,B**) and behavioural (**C,D**) alterations in arthritic animals. (**A**) Weight gain (g) between week 3 and week 0. (**B**) Global inflammation score showing a strong inflammation in arthritic animals (from 0 to 60). (**C**) Reduction of the threshold of static mechanical hypersensitivity, suggesting a bilateral mechanical hypersensitivity in arthritic rats. (**D**) Measure of the vocalizations evoked by bending of the hind paw (bending score from 0 to 5) shows a strong bilateral hypersensitivity to paw mobilization. N = 9 per group. ***p < 0.001 (U-Mann Whitney test).

At three weeks post-induction of arthritis, which is a time point known to be characterized by chronic inflammatory pain^31,48^, arthritic animals had a significant reduction in body weight gain, which is an indirect marker of sustained spontaneous pain (while control animals gained an average of 136.00 g ± 11.76, arthritic animals only gained 49.44 g ± 11.59 g, p = 4.10^−5^, U-Mann Whitney, Fig. 2A). The Inflammation score was significantly increased in arthritic rats versus controls (p = 4.10^−5^, U-Mann Whitney), with a median score for each group of 22 and 0, respectively (Fig. 2B).

Measurement of the static mechanical threshold showed a bilateral static mechanical allodynia in both the right and left hind paws in arthritic animals compared to the control group (respectively p = 8.10^−4^ and p = 3.10^−4^, U-Mann Whitney, Fig. 2C) and a significant increase in the foot-bend score of both right and left ankles in arthritic animals (p = 4.10^−4^ for both), suggesting increased mechanical hypersensitivity to ankle movement.

In summary, these results confirm that, as observed in the clinical condition, arthritic animals displayed a consistent but variable peripheral inflammation and mechanical hypersensitivity (static and dynamic) of both inflamed hind paws.

### Alterations in brain FC linked to long-term inflammatory pain

#### Seed-based analysis

Our first analysis of the potential FC changes associated with pain maintenance was performed using ‘seed-based’ analysis. In each imaged plane (see Materials and Methods), we tested various combinations of regions of interest (ROIs, Supplementary Table 1) to determine whether the connectivity was altered (Supplementary Table 2). These ROIs were chosen based on the definition of structures involved in pain processing^49,50^. A more thorough analysis was later performed using multiple ROIs defined using the Paxinos rat brain atlas (Fig. 4).

**Figure 3:**
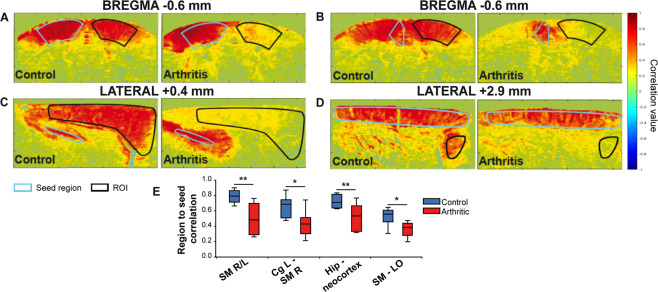
Seed-based analysis of the FC alterations in arthritic animals. (**A**–**E**) are typical examples of the correlation between a seed region (ROI delineated in cyan) and the pixels in the imaged plane. The Pearson correlation was then computed in the ROI (delineated in black). The results in (**A**–**E**) and their quantification in N = 9 animals per group show that arthritic animals display a reduced bilateral connectivity in the somatomotor (p = 0.002, **A**), cingulo-cortical (p = 0.02, **B**), cortico-hippocampal (p = 0.01, **C**) and cortico-orbital (p = 0.02, **D**) networks. (**F**) Boxplots of the correlation coefficients for each group of rats (N = 9 per group) and each pair of ROIs (SM = Somatomotor cortex, Cg = Cingulate cortex, Hipp = Hippocampus, LO = Lateral orbital cortex). *p < 0.05 and **p < 0.01, Welch’s test, or Mann-Whitney test in case of non-normality after Fisher transformation, followed by Benjamini-Hochberg’s correction for multiple comparisons.

**Figure 4:**
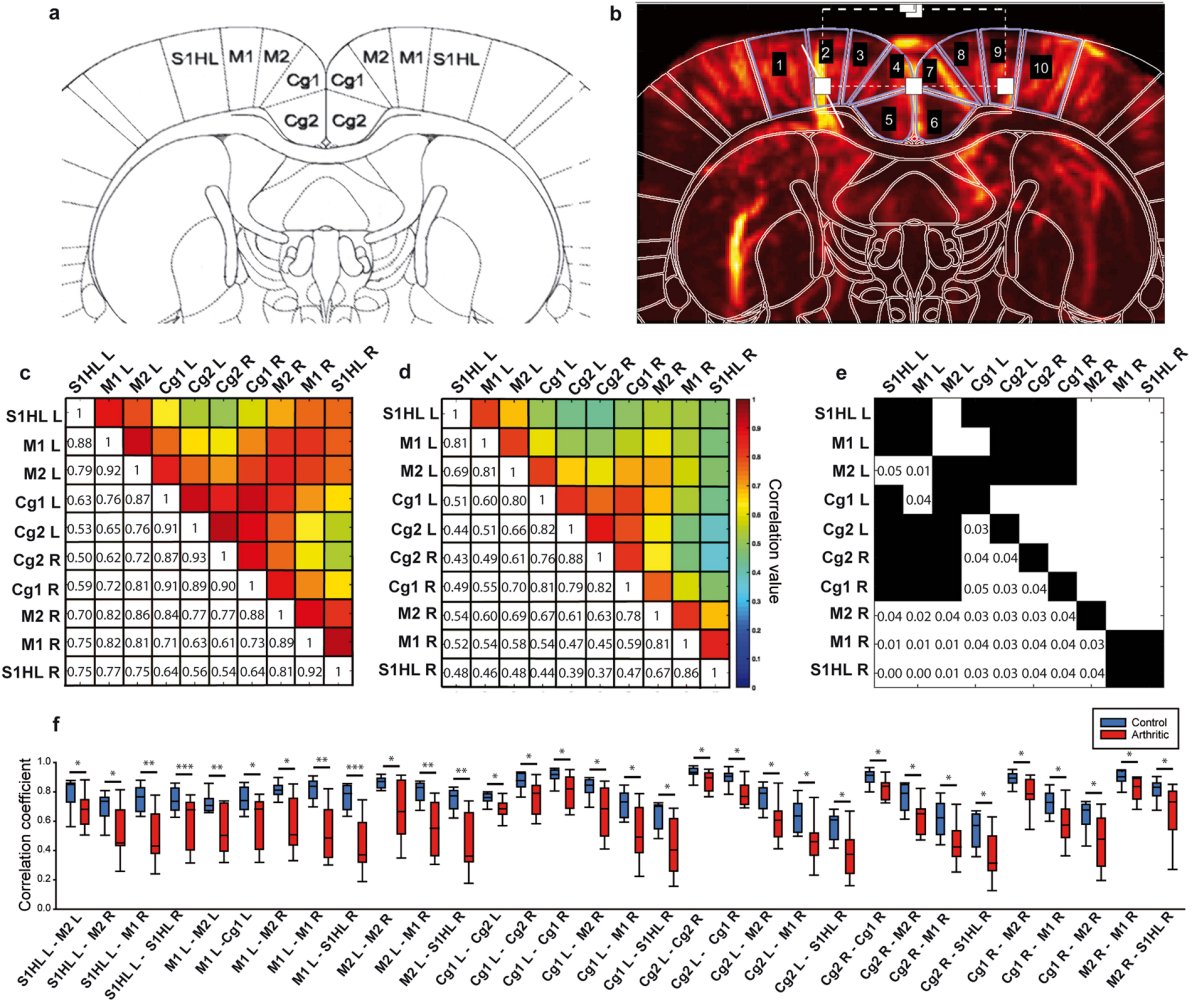
Correlation matrix analysis of the FC on the −0.6 Bregma plan of the rat brain atlas. (**a**) Plane of interest of the Paxinos rat brain atlas (Bregma −0.6 mm) and corresponding ROIs (S1HL, M1, M2, Cg1, Cg2 left and right for each), with permission from Elsevier. This image is modified from ‘Rat Brain in Stereotaxic Coordinates’. Paxinos &Watson, Academic Press, San Diego 3rd, (1997). (**b**) Doppler image of the rat brain with the corresponding mask of the rat brain atlas presented in (**a**). (**c,d**) Averaged Pearson correlation matrix for each of the 9 rats in the control and arthritic groups. (**e**) Matrix of significance of the differences between c (control) and d (arthritic) matrices. White squares represent the pairs of ROIs with a significant alteration in FC between the two groups, with corresponding p-values. (**f**) Boxplot representation of each pair with a significant FC alteration. N = 9 per group. *p < 0.05, **p < 0.01 and ***p < 0.001, Welch’s test, or Mann-Whitney test in case of non-normality after Fisher transformation, followed by Benjamini-Hochberg’s correction for multiple comparisons.

As summarized in Supplementary Table 2, after four weeks of peripheral inflammation, we observed strong alterations of FC in a small number of ROIs. These alterations were: reduced left and right sensorimotor connectivity (at Bregma −0.6 mm, p = 0.002, Fig. 3A), reduced connectivity between the left cingulate cortex and right sensorimotor cortices (p = 0.02, Fig. 3B), reduced connectivity between the hippocampus and the neocortex (p = 0.01, Fig. 3C, imaged in a sagittal plane at Lateral +0.4 mm) and decreased connectivity between the sensorimotor cortex and the lateral orbital (LO) cortex in arthritic rats (p = 0.02, Fig. 3E, sagittal plane at Lateral +2.9 mm). In conclusion, our observations suggest a profound reduction in the FC in a part of cortico-cortical networks in arthritic animals.

#### Correlation matrices analysis

This FC analysis was further investigated by defining smaller ROIs, as defined by the rat brain atlas^35^. The main advantage of this approach compared to the previous one is the more precise analysis of FC alterations in functionally defined areas, especially within areas that were defined as one ROI in the seed-based analysis (i.e. sub parts of the primary sensory cortex or of the sensory-motor network). However, due to the large number of tests performed and the associated multiple comparison corrections, only very strong changes in connectivity can be detected using such an approach.

The correlation matrices for the five planes were computed for each rat in the control and arthritic groups. The statistical analysis revealed significant alterations exclusively in the medial coronal plane Bregma −0.6 mm (Supplementary Table 3, Fig. 4e). The significant changes were consistently reductions in FC, as detailed in Fig. 4f. This reduced connectivity among the ROIs were: right-left (R-L) S1HL (Primary sensory cortex, Hind Limb part), S1HL-M1 (Primary motor cortex), S1HL-M2 (Secondary motor cortex), S1HL-Cg1 (Primary cingulate cortex), S1HL-Cg2 (Secondary cingulate cortex), R-L M1, M1-M2, M1-Cg1, M1 and Cg2, R-L M2, R-L Cg1, R-L Cg2, Cg1 and Cg2, Cg2 and M2 (Fig. 4c–f).

Analysis in other planes in arthritic rats did not reveal any significant changes, suggesting that the changes observed in these other planes using the seed-based approach may not be statistically powerful enough (because of the necessary correction for multiple testing) to be revealed using the correlation matrix analysis.

These results are confirming previous results, i.e. an alteration of the FC in some subparts of the SM network and further describe in detail the strengths of this reduced connectivity in all the ROIs of the SM network. However, it has to be noticed that a large part of this network, the parts not dedicated to the hind paws, is not affected by these changes.

#### Dynamical FC analysis

While the seed-based and correlation matrix analyses measure the averaged correlations of hemodynamic fluctuations between two brain areas over time, several studies have shown that FC has an intrinsic dynamics^2,7,8,51^, and such dynamics are interesting to study because, for instance, they are altered in neurological disorders^2,52^. Following this postulate and previous demonstrations by other teams of various ‘states of the brain’^9,53,54^ using fMRI, we i) analyzed the dynamics of the fUS signal phase (its temporal dynamics in arthritic and control animals), ii) extracted the brain states from the dynamics of functional connectivity using unsupervised clustering (k-means algorithm) and iii) finally studied their possible alterations in long-term inflammatory pain. This analysis was applied to all imaged planes, using the ROIs of the correlation matrix analysis.

**Figure 5:**
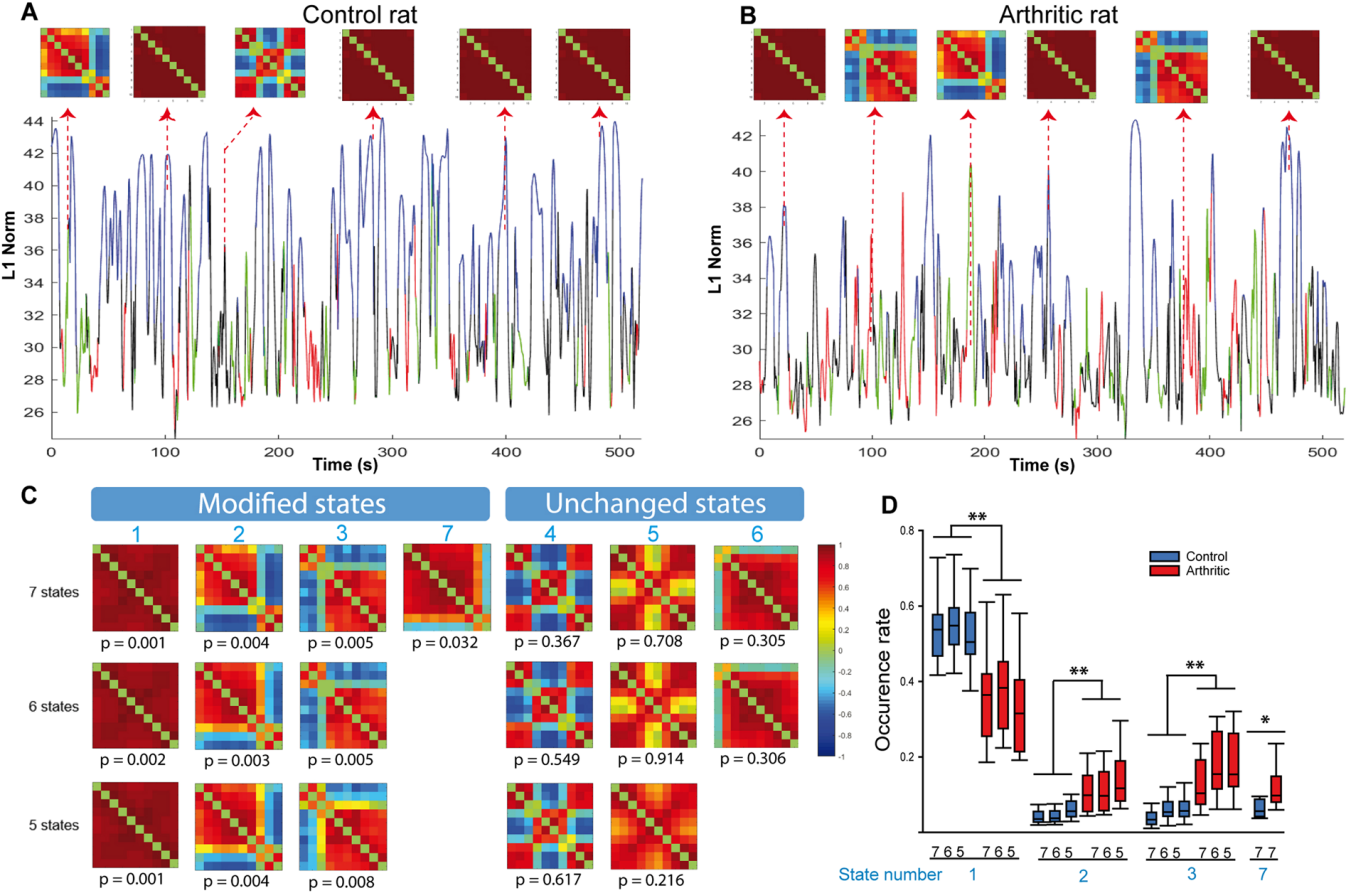
Dynamic brain state changes in control and arthritic rats imaged in the plan Bregma −0.6 mm. (**A**,**B**) Time course of the L1 norm of the phase-locked matrix of a control and an arthritic rat, respectively. Inserted matrices show brain phase patterns at different time points. As previously described by Barttfeld *et al*.^9^ the time series show an exploration of brain states over time. (**C**) Decomposition into brain states 5, 6 and 7 obtained by unsupervised k-means clustering of the phase matrices. The ROIs are the same as in Fig. 4. The decomposition produces robust results, with the matrices obtained for k = 5, 6 or 7 states being very consistent. The p-values written under each matrix show that for four states (states 1, 2, 3 and 7), the occurrence over time is significantly altered between controls and arthritics. (**D**) Box plots presenting the occurrence rate of each of the four different states depending on the decomposition value k. The first modified state appears significantly more often over time in the control group than in the arthritic group. The other three states appear less frequently over time in the control group than in the arthritic group. While arthritic animals spend less time in the state 1, they spend significantly more time in the states 2 and 3. N = 9 per group. *p < 0.05, **p < 0.01 (U Mann Whitney) indicate significant differences between the control and arthritic groups for a given brain state (state number in blue), and a given k = 5, or 6 or 7.

A time course of phase-locked matrices was computed for each rat in the control and arthritic groups to obtain an overall perspective of the phase over time. Figure 5A,B shows two typical examples of time courses in the same imaging plane (and the same ROIs as in Fig. 4) in one control and one arthritic rat and the phase-locked matrices at six random time points.

Second, an unsupervised analysis of the phase-locked matrices of the two groups (controls and arthritics) was performed for three different values of the number of clusters k. Very reproducibly, this analysis showed that decomposition in either five, six or seven states robustly defined five similar states of the brain (Fig. 5C). These observations highlight the strength of the algorithm and its robustness. Because the main goal of this study is to understand the brain function alterations in chronic pain diseases, we measured the rate of occurrence of each of these k brain state matrices (k = 5, 6 or 7) during the ten minutes of acquisition for all animals (control and arthritic rats). Statistical analysis showed that, while they all appear in both groups of rats, four states had significantly different probabilities of occurrence (Fig. 5) depending on whether the rat was a control or an arthritic (p < 0.05).The arthritic animals spent less time (reduced occurrence rate) in the dynamic state 1 (p < 0.05), a dynamic pattern of FC, where the signals of the whole SM network and DMN oscillate in synchrony (cosinus of the phase at ‘1’, red color). On the other hand, arthritic animals spent more time (increased occurrence rate) in the state 2 and phase 3, which are, respectively patterns characterized by anti-correlated signals (cosinus of the phase at ‘−1’, blue color) between the right somatomotor cortex and the rest of the SM, (i.e. the contralateral SM and the cingulate cortex), while state 3 is the opposite situation: signals from the left somatomotor cortex is in opposing phase with the right SM and the cingulate cortex., (p < 0.05) (Fig. 5D). States 2 and 3 are therefore two dynamic patterns, where a part of the synchrony of the whole SM network is maintained, but the primary motor and primary sensory cortex of each side anti-correlated with it.

States 4 (state where the primary and secondary cingulate cortices are in opposing phase with the right and left SM), 5 (state characterized by an asynchrony between the secondary cingulate cortex and the rest of the SM) and state 6 did not exhibit any significant occurrence rate differences between the two groups (Fig. 5 and Supplementary Fig. 1). states 2, 3 and 7 were more frequent in arthritic animals than in controls (p < 0.05) (Fig. 5D).

Such changes in brain states were observed also in two other imaging planes including a more posterior part of the SM network and a part of the reward circuit: coronal Bregma −3.84 mm and sagittal Lateral +2.9 mm, respectively, showing an increased (p < 0.05) and a decreased (p < 0.05) occurrence rate in arthritic animals compared to controls (Supplementary Fig. 2).

These results suggest that it is possible to measure alterations of dFC resulting from brain function remodeling due to chronic pain in anesthetized animals, using fUS. In addition, they indicate strong and significant alterations of the dynamic of some subnetworks in arthritic animals.

### Correlations between the FC alterations and behavioral changes

One of the challenges in the study of neurological disorders is that, despite the power of neuroimaging to image the brain during a task (functional neuroimaging) or at rest (resting-state FC), translating these network alterations to changes in animal behavior remains a complex issue. This is why the second part of this study aimed to identify possible links between the alterations of specific brain networks (using analyses of: the seed-based, correlation matrix and dFC) and the different physiological/behavioral alterations, namely changes in weight gain, inflammation, mechanical hypersensitivity (static mechanical allodynia or dynamic allodynia induced by the mobility of the ankle). The hypothesis behind this approach is that, beyond the overall group effect and individual variability in all these behavioral parameters, the correlation among all these individual values and the individual FC scores might reveal clear links between some aspects of pain (sensory-discriminative, emotional or cognitive) and the observed brain network alterations.

**Figure 6:**
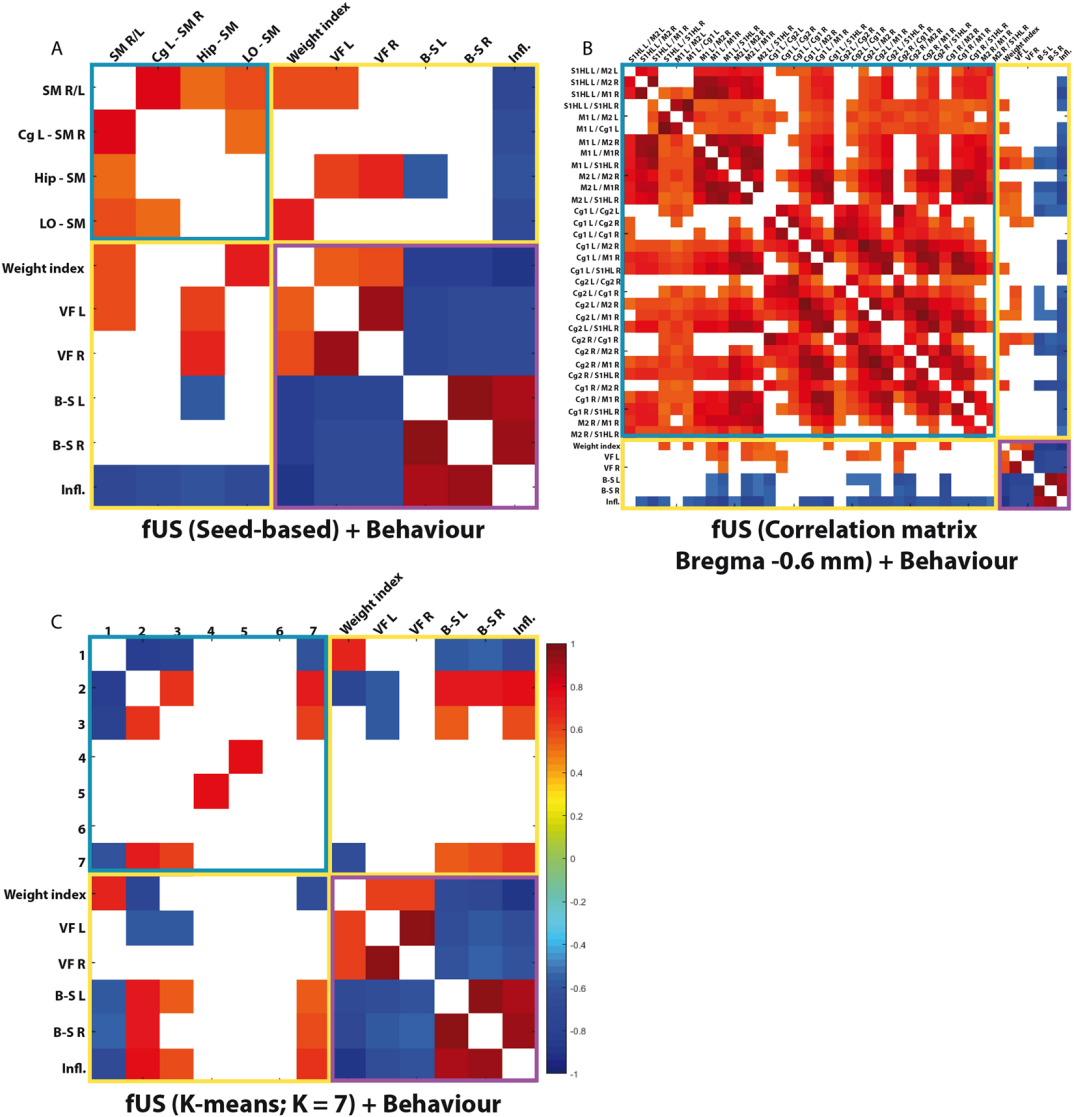
Statistically significant correlations between individual physiological or behaviour alterations and altered FC (**A,B**) or brain states (**C**). (**A,B**) Spearman correlation of the 4 and 32 significant results from the seed-based (Bregma −0.6 mm and the two lateral planes, as shown on Fig. 3) (**A**) or correlation matrix (Bregma −0.6 mm, as shown on Fig. 4) analyses (**B**), respectively, with behaviour. (**C**) Correlation of behaviour with the occurrence probability of each state from the k-means decomposition into 7 states. For each type of analysis (seed-based, correlation matrix and FC dynamics), we correlated all individual results (from all control and arthritic rats) with the individual behavioural data. Following a Spearman correlation computation, the results show only the statistically significant correlations (p < 0.05). The associations between FC/brain states and FC/brain states are outlined with a blue square; correlations between FC/brain states and behaviour is indicated by a yellow square; and finally, associations of behaviour with behaviour are outlined with a purple square. This analysis shows specific positive or negative correlations between either brain states or changes in restricted FC networks, suggesting that a link exists among FC/brain states alterations and the various aspects of pain in arthritic animals. N = 9 per group.

For each type of analysis (seed-based, correlation matrix and dynamical connectivity), we correlated all individual results with the individual behavioral data. Significant correlations after Benjamini-Hochberg multiple comparison correction (p < 0.05) were observed between alterations of FC networks/’brain states’ between themselves (blue outline, Fig. 6A–C), alterations of FC networks/’brain states’ with subsets of pain behavior and FC/’brain states’ alterations (yellow outline, Fig. 6A–C).

#### Seed-based vs behavior

The correlation matrix of the seed-based analysis with behavior (yellow outline, Fig. 6A) showed that the connectivity alterations of all the significantly functionally altered pairs were strongly and negatively correlated with inflammation score (Fig. 6A). This means that the reduction of connectivity between these areas is linked with increased inflammation score. The changes in FC between the right and left somatosensory cortices and between the LO and somatosensory cortex both showed significant positive correlations with weight index, meaning that the FC alterations between those areas are linked with decreased weight gain/weight loss in arthritic rats as compared with controls. The most remarkable result was the very strong correlation between the change in hippocampus-somatosensory cortex connectivity and behavior. Indeed, the decrease in connectivity of this pair significantly and positively correlated with static mechanical sensitivity and this decrease also significantly and negatively correlated with the bending test of the left paw (Fig. 6A, Supplementary Table 4).

#### Correlation matrices vs behavior

The Spearman correlation matrices of the FC alterations in pairs of ROIs defined using the rat brain atlas showed a large number of significant correlations with behavioral indexes (Fig. 6B). First, in agreement with the previous seed-based analysis, inflammation score showed a consistently significant and negative correlation with the evolution of the FC reduction (the ROI pairs being the ones that showed significant FC alterations, see Fig. 4E). Second, weight gain positively correlated with several FC pairs. All other behavioral tests correlated far less with the connectivity alterations. Specifically, the couples that correlated most strongly with the behavioral data were the M1 L/R, the M1 L/S1HL R, the M2 L/M1 R, the M2 L/S1HL R, the Cg1 and Cg2 L/R, the Cg2 L/M1 R, the Cg2 and M2 R, and the Cg1 and M2 R (see Fig. 6B and Supplementary Table 4). They almost always significantly correlated with weight gain, FC analysed in seed-based or matrix and dynamic mechanical hypersensitivity and inflammation score.

#### FC dynamics vs behavior

Calculation of the Spearman correlation between the occurrence probabilities of each brain state and the behavioral data showed that only state 1, which showed a higher occurrence rate in the controls than in the arthritics, was significantly correlated with weight gain, the bilateral bending test, and inflammation score (Figs. 6C and Supplementary Table 4). Spearman correlations for states 2 and 7 showed contrasting results; these states, which were all more present in the arthritics than in the controls, were significantly correlated with weight gain, the Von Frey test of the left paw, the bilateral bending-stretching tests and inflammation score. A similar tendency was observed for state 3 but at a lower level of significance.

These different correlations of the occurrence probabilities of states 1, 2, 3 and 7 with the behavioral data suggest that a link exists among brain states alterations, arthritis and the various aspects of pain.

### Diagnostic power

**Figure 7:**
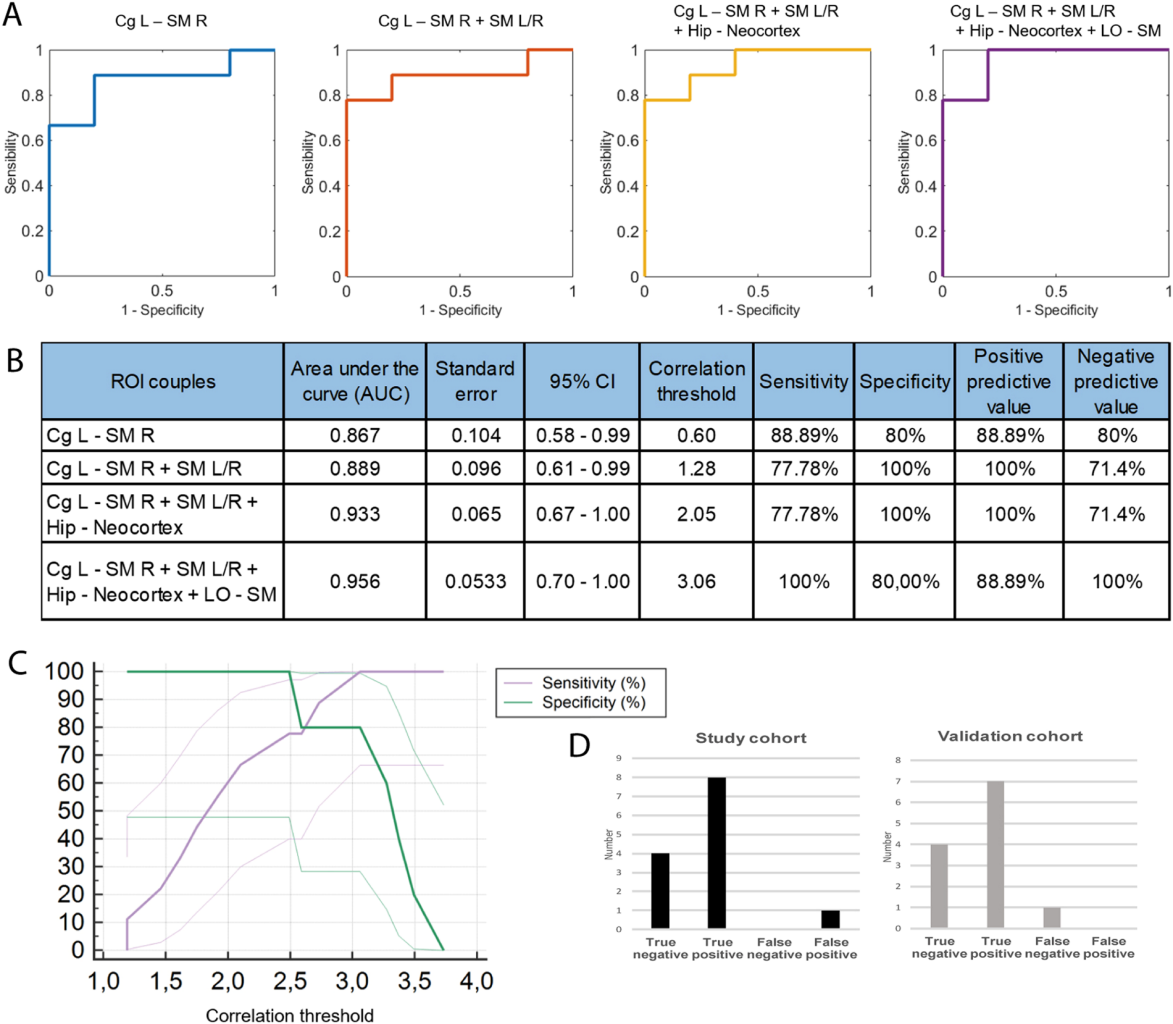
ROC curves derived from the FC data using the seed-based analysis of four statistically significant pairs of ROIs imaged at different planes in control and arthritic rats. The figure shows the increase in sensitivity (or true positive rate) and specificity (or true negative rate) when discriminating between the ‘control’ and ‘arthritic’ conditions obtained when the number of ROI pairs is increased. The individual pairs of ROIs computed were (i) Cg L and SM cortex R (imaged at Bregma −0.6 mm); (ii) Cg L and SM cortex R + SM cortex L/R (imaged at Bregma −0.6 mm); (iii) Cg L and SM cortex R + SM cortex L/R (imaged at Bregma −0.6 mm) + hippocampus and neocortex (imaged at Lateral +0.4 mm); (iv) Cg L and SM cortex R + SM cortex L/R (imaged at Bregma −0.6 mm) + hippocampus and neocortex (imaged at Lateral +0.4 mm) + LO and SM cortices (imaged at Lateral +2.9 mm). The use of four FC pairs leads to an AUC of 0.975 and an optimal discrimination between arthritic and control rats with 100% sensitivity and 88.9% specificity (**A,B**). (**C**) Plotting of the sensitivity and specificity obtained using these four FC pairs and the correlation threshold obtained. (**D**) Cross validation of this approach using a separate cohort of animals (‘Validation cohort’), using the correlation threshold previously defined, showed 87,5% sensitivity and 100% specificity using the same four FC pairs of ROIs. (**D**) shows a similar magnitude of false negative and positive results.

The third part of our study focused on the selectivity and sensitivity of the FC alterations in somatomotor networks to discriminate arthritic animals from controls. Receiver Operating Characteristic (ROC) curves, which are routinely used in medical research to evaluate screening tests, were computed using FC data from our seed-based analysis. Using a first cohort of animals, ROC curves summarizing the sensitivity/specificity power were generated using composite biomarkers containing an increasing number of FC regions (corresponding to ROI pairs in the somatomotor cortices at Bregma −0.6 mm that displayed significantly different FC in arthritic versus control animals). The first pair (blue line, left cingulate and right somatomotor cortex) by itself has substantial discrimination power, with an Area Under the Curve (AUC) of 0.867 (95% CI 0.58–0.99, p < 0.0001, Fig. 7B). A correlation threshold of 0.60 was associated with 88.9% sensitivity and 80% specificity in discriminating between control and arthritic rats. This corresponds to a 88.9% Positive Predictive Value (PPV) and a 80.0% Negative Predictive Value (NPV). The successive addition of three other significant ROI pairs further increased this percentage and the AUC. Using a four couples logistic model, the AUC reached 0.956 (95% CI 0.70–1.0, p < 0.0001, Fig. 7B). The optimal correlation threshold was obtained for the four pairs (correlation threshold of 3.06) was associated with 100% sensitivity and 80.0% specificity in discriminating control and arthritic rats, leading to a 88.9% PPV at 100.0% NPV (Fig. 7B,C).

In a second step, this observation was then backcrossed using a second cohort of animals (“Validation cohort”, Fig. 7D). Using the threshold defined in the first cohort of animals (“Test Cohort”), we obtained 87,5% sensitivity and 100% specificity in the separation of both groups of animals in this new cohort (Fig. 7D), which is slightly lower than the first cohort, but remains very high for a robust discrimination between control and arthritic animals only based on their FC alterations.

In conclusion, alterations of FC in this sub-part of the somatosensory network, in this animal model, is strong and reproducible enough among all arthritic animals to be used as a biomarker of cortical changes associated with arthritis in rats.

## Discussion

Study of resting state fMRI functional connectivity has provided a unique tool for the study of the intrinsic functional organization of the brain, but also for the measure of network dysfunction in pathological conditions. A growing literature described functional and structural alterations of the brain in multiple chronic pain syndromes^55,56^. Whereas extensive results have been obtained thanks to fMRI resting state imaging in clinics, pre-clinical research remains hampered by MRI technological limitations. This study proposed a new ultrasound neuroimaging approach to reproducibly study in depth alterations of FC networks and brain states in such a preclinical model. We show that, despite the presence of anesthesia, arthritis is associated with potent alterations of the functional connectivity and brain states in some specific networks.

### Neurobiological relevance of FC alterations in the somatomotor cortex of arthritic animals

In this study, after selection of five dedicated imaging planes, we observed that the connectivity between the hippocampus and the neocortex, but also between the lateral orbital cortex (LO) and the neocortex undergo significant alterations in arthritic animals. Among intrinsic networks, the default mode network (DMN) is a key network altered in chronic pain^4,40,57–62^. Normally active at rest, it is deactivated during task or stimulus exposure, including acute pain^1,63^. A large literature in both chronic pain patients and animal models shows an abnormal deactivation of the DMN during task- or pain-related stimulations^61,64,65^ and exhibit abnormal DMN resting state functional connectivity associated with pain intensity and rumination^4,58,59^. Despite the fact that our technique does not allow us to image the entire DMN, imaging in the sagittal plane lat +0.4 mm, where most of the medial areas of the DMN are located^66^, revealed a reduced connectivity between structures of the DMN, a feature consistent with previous observations in patients suffering from arthritis^4,15,40,61^.

Importantly, the strongest changes of FC observed (reduction) was observed in a subsection of the somatomotor network. These changes were restricted in the somatomotor cortex to the region dedicated to the inflamed paw. This specific and localized plasticity could be due to the increased electrophysiological activity previously described in this model/pathology^67,68^. These results are consistent with a previous study showing a widespread alteration in the activity of the somatosensory network and major change in the functionality of the cingular-somatosensory network after 1 week of inflammation of the whisker pad^69^. These observations are also consistent with a recent study using a different modality (EEG)^70^ in an animal model of neuropathic pain, demonstrating that during the course of chronic pain installation, brain networks become functionally altered.

Importantly, while the aforementioned study was performed in conscious animals using EEG^70^, ours used fUS imaging in (moderately) anesthetized animals. Whereas the presence of anesthesia (even at low dose) may have reduced the FC signal we sought to measure, as previously observed by other authors^71,72^, the results obtained in our study suggest that these measured effects were strong enough to not be dampened by anesthesia. However, it cannot be excluded that stronger changes might be found in conscious and freely moving animals and this will be the topic of future studies.

### Alterations of some brain states estimated using k-means clustering in arthritic animals

fUS signal fluctuations over time can be analyzed dynamically to define various brain states^53,54^. In this article, we demonstrate for the first time that dynamic functional connectivity assessed by ultrasound can provide quantitative and robust information on the alterations of brain networks. We chose to use the phase of the fUS Doppler signal, i.e., the patterns of delays between signals from various ROIs^10,47^. Because the element that clustered in our analysis (through k-means) is the phase of the signal, we can hypothesize that each ‘brain state’ represents alternating modes where the ROIs have a common synchronism (cosinus of the phase at ‘1’, red color), have an anti-synchronism (cosinus of the phase at ‘−1’, blue color), or are delayed (any other combination).

The second major discovery described in this study is that in the brain-states of dFC, one brain state (# 1, which is the most occurrent and is a dynamic pattern of FC, where the signals of the whole SM network and the DMN hub cingulate cortex oscillate in synchrony) was significantly less frequently observed in arthritic animals, whereas on the other hand, two other states (# 2, 3, that are two dynamic patterns, where the primary motor and primary sensory cortex of each side anti-correlate with the rest of the SM network) occurred more frequently in these animals. The lower occurrence of the brain state #1 in arthritic rats shows that the synchronism between the somatosensory, motor and cingulate cortices is highly affected by the pathology. This very interesting result, which is in agreement with a recent description of alterations of the cingular-somatosensory interplay in inflamed animals^69^, opens the way to a new approach to understand brain functions and their alterations in chronic pain states.

Interestingly, states 2 and 3 are two dynamic patterns, where the synchronicity of the SM network is interrupted: a block formed by the primary motor and primary sensory cortices (right and left) anti-correlate with the rest of the SM network. As both the right and left hind paws are inflamed in arthritic animals, it can be hypothesized that these anti-correlations in the primary motor and primary sensory motor dedicated to the inflamed paws are due to the ongoing sensitization and tonic discharge of peripheral nociceptors, ultimately activating the primary sensory cortex and leading to this breach of synchronism. In other words, this breach of synchronism observed in states 2 and 3 might be an indirect measure of the spontaneous firing of nociceptors, a feature known to induce spontaneous pain.

The activation in the primary motor cortex following peripheral stimulations is however unexpected. Several reasons could explain this surprising observation: i) In our study, similarly to other authors, we defined the ROIs not functionally, but by applying blindly the Paxinos atlas. It is known that the ROIs provided by the atlas are relatively approximative and their definition keeps being improved by the authors. In addition, we previously observed that the hemodynamic response induced by noxious stimulations is located in the area S1HL described by the atlas^6^, but spreads in a part of the primary motor cortex. Looking closely at the activation observed by other authors using fMRI, such observation can also not be ruled out^73–75^. Ii) In addition, the map may not consider the complex functional architecture of the primary sensory cortex and its plasticity, as recently unraveled^76^. Finally, the subdivisions of the S1HL that received the information from tactile versus nociceptive information may not be the same, as observed in monkeys^77^. As a consequence, it is very likely that the ROI that show anti-correlation with the rest of the matrix in the states 2 and 3 correspond functionally (using the sensitivity of fUS imaging) to the area dedicated to the inflamed paw, where an increased electrophysiological activity was previously described by previous teams^67^. In conclusion, analysis of dFC through analysis of brain states, that was recently used to characterize the dynamic patterns of brain signal in link with consciousness^10^, is a promising alley that will shed light on the dynamics of the chronic pain connectome. Its use on fMRI resting state data of cohorts of various chronic pain patients would be very useful to evaluate its potential translational application. Its use on fUS resting state data is here shown to provide reproducible and robust information on the alterations of brain functions in chronic pain states for animal models.

### Alterations of FC networks and their links with physiology and behavior

Currently, studies of the physiology and pathophysiology of resting-state networks represent the core of numerous studies. The study of the structural networks underlying FC, Brain Connectomics, has also become an emergent field within neuroscience^78^. While these elements are two pieces of the same puzzle and naturally fit together to provide complementary information on brain networks and their alterations, far less is understood about the link between functional alterations in brain networks and behavioral changes. Previous studies have directly addressed this connectomics/behavior link^15,41,79,80^, which is an important component of the present study. Using individual correlations between FC alterations (occurrences of state of dFC) and several aspects of clinical/pain behavior in arthritic and control animals (either mechanical sensitivity, inflammation score or differences in weight gain), our results revealed a very small number of brain areas with significant links to pain behavior, suggesting the presence of subtle and specific links between these brain alterations and behavioral changes.

Using both the seed-based approach and the correlation matrix analysis, we evidenced an important and significant correlation between somatomotor network alterations and weight gain (reduction in weight gain is an indirect index of the overall burden of the disease on the animal’s physiology) or mechanical hypersensitivity. The two types of analyses produced consistent and complementary results, suggesting strong and specific results. We hypothesize that alterations of the somatomotor network represent one of the mechanisms underlying the discriminative aspect of persistent pain.

The most striking correlation result is perhaps the positive correlation in FC alterations between the LO cortex - SM cortex and body weight gain, suggesting a decreased FC in arthritic animals with decreased body weight gain. The FC of this network was also anti-correlated with the inflammation score. The LO is the equivalent in humans of the ventrolateral orbital cortex (VLO), which is part of the prefrontal cortex, known to be linked to pain, memory, and emotion. It has been suggested that the VLO is involved in the processing of both pain sensation and pain affect^81,82^. Indeed, surgical lesion of the orbital cortex in patients has been shown to provide relief from chronic pain^83^, and pre-clinical studies have demonstrated that the VLO is involved in modulating nociceptive responses via both dopaminergic and opioid receptors. This last effect is mediated through activation of a descending pathway from the periaqueductal gray to the spinal cord^84^. Our hypothesis is that in arthritic rats, the functionality of this area linked to the emotional and cognitive aspects of pain is altered, and as a consequence, such changes may not be linked to the sensory aspect of pain but rather to cognitive aspects of pain, such as coping with persistent pain.

Another brain area, whose FC is significantly altered (Hippocampo-Cortical connectivity) and whose changes are linked to changes of behavior, is the hippocampus. Hippocampus is known to be involved in memory and spatial recognition. It has been depicted as a key mediator of aversive drive and affect of pain for a long time, because lesions of the hippocampus alter the perception of noxious stimuli and partially alleviates pain^85,86^. Regarding its role in the pathophysiology of chronic pain, several studies have underlined its implication in the mechanism of pain “chronification”. For instance, increased FC between hippocampus and striatum was observed in rats with a spared nerve injury^42^. Interestingly, similarly to our observations, they observed a decreased FC between the hippocampus and the somatomotor cortex, suggesting that such changes might be a common feature to several models of persistent pain. Comorbid pathologies, such as anxiety and depression are known to be prevalent when pain becomes chronic^87^. Hippocampus is also known to be indirectly linked to regulation of mood disorders. Rodents with a reversible neuropathic injury continued to exhibit depressive- and anxiety-like behaviours, in correlation with a decreased hippocampal neurogenesis^88^. Also, Duric and McCarson found that persistent inflammatory pain in rats induces hippocampal neurogenesis and altered hippocampal morphology, as does chronic stress^89^. It is thus possible that the changes we imaged in arthritic rats, i.e. the reorganization of the processing within the hippocampus and the cortex, are, as previously described in patients with chronic back pain patients^90^, a contributor to the transition from acute to chronic pain and possibly to comorbidities.

In conclusion, this article provides a robust and quantitative multimodal analysis of the alterations of brain functions through the analysis of altered dynamic functional connectivity (analyzed in k-mean clustering). Combination with individual behavioral scores showed a clear link between these impairments and some aspects of animal’s clinical and/or pain state. This approach paves the way to a quantitative analysis of dFC evolution during the course of diseases in pre-clinical models. The combined time courses of FC alterations and behaviour could potentially provide new translational insights on relevant targets for human studies, as FC can easily be assessed by resting state fMRI in clinical environment.

## Supplementary information

Supplementary dataset.
