# Supplementary material for: Ultrafast ultrasound imaging pattern analysis reveals distinctive dynamic brain states and potent sub-network alterations in arthritic animals
Source: Sci Rep. 2020 Jun 26;10:10485. doi: 10.1038/s41598-020-66967-x (PMC7320008; doi:10.1038/s41598-020-66967-x)
Supplement: Supplementary file 1 — Supplementary dataset. [file 41598_2020_66967_MOESM1_ESM.pdf]

Ultrafast ultrasound imaging pattern analysis reveals distinctive dynamic brain states and potent sub-network alterations in arthritic animals

Line Rahal[1,2,*], Miguel Thibaut[1,*], Isabelle Rivals[3], Julien Claron[2], Zsolt Lenkei[1,4], Jacobo D. Sitt[5], Mickael Tanter[2,†] and Sophie Pezet[1,2,†]

List of the supplementary materials below:

Supplementary Figure 1

Supplementary Figure 2

Supplementary Figure 3

Supplementary Table 1

Supplementary Table 2

Supplementary Table 3

Supplementary Table 4

Supplementary figure 1

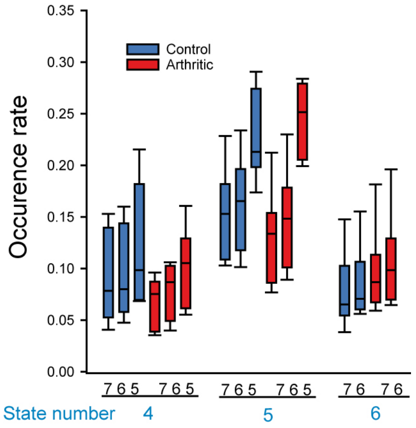

Supplementary figure 2

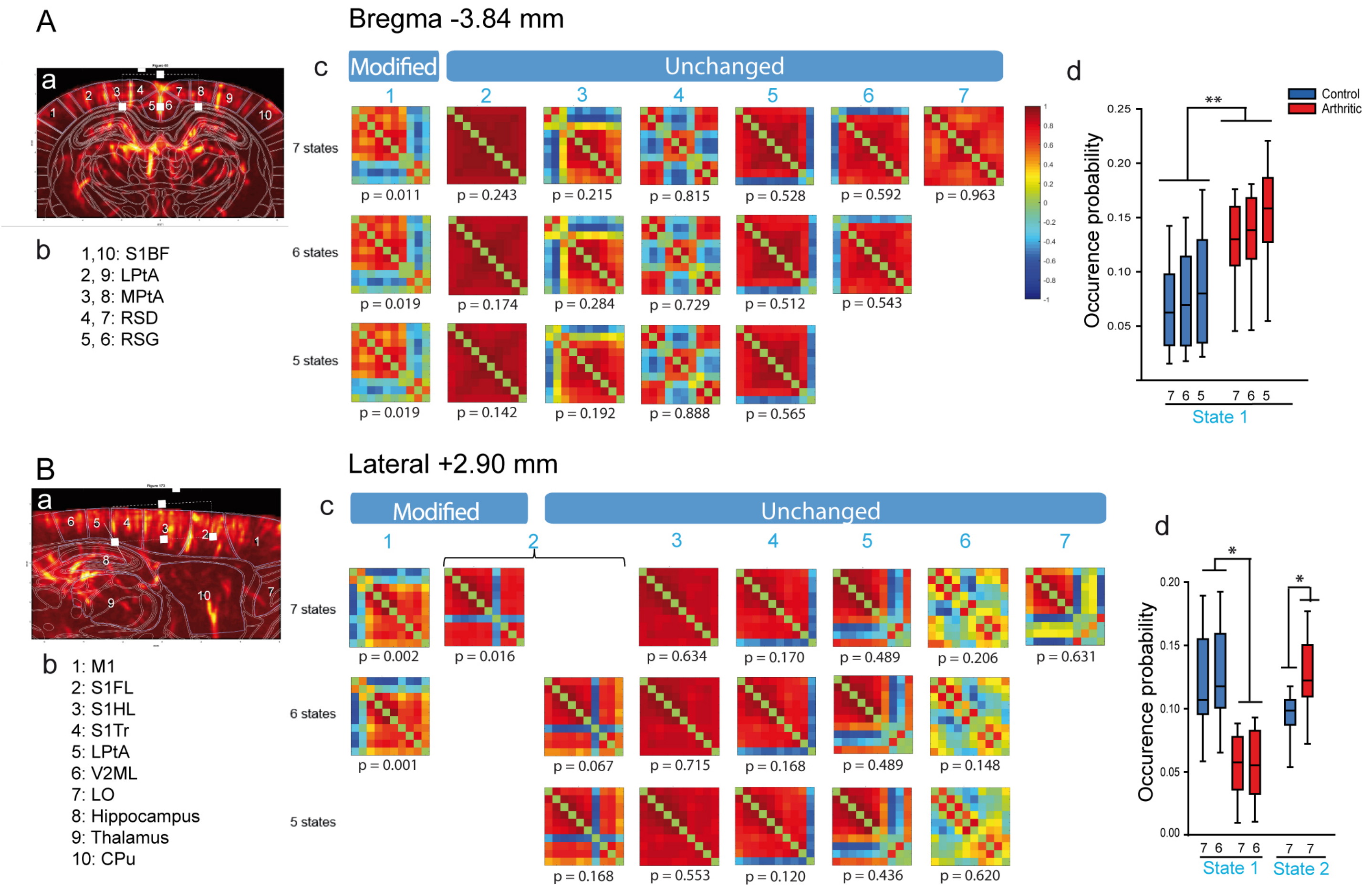

Supplementary figure 3

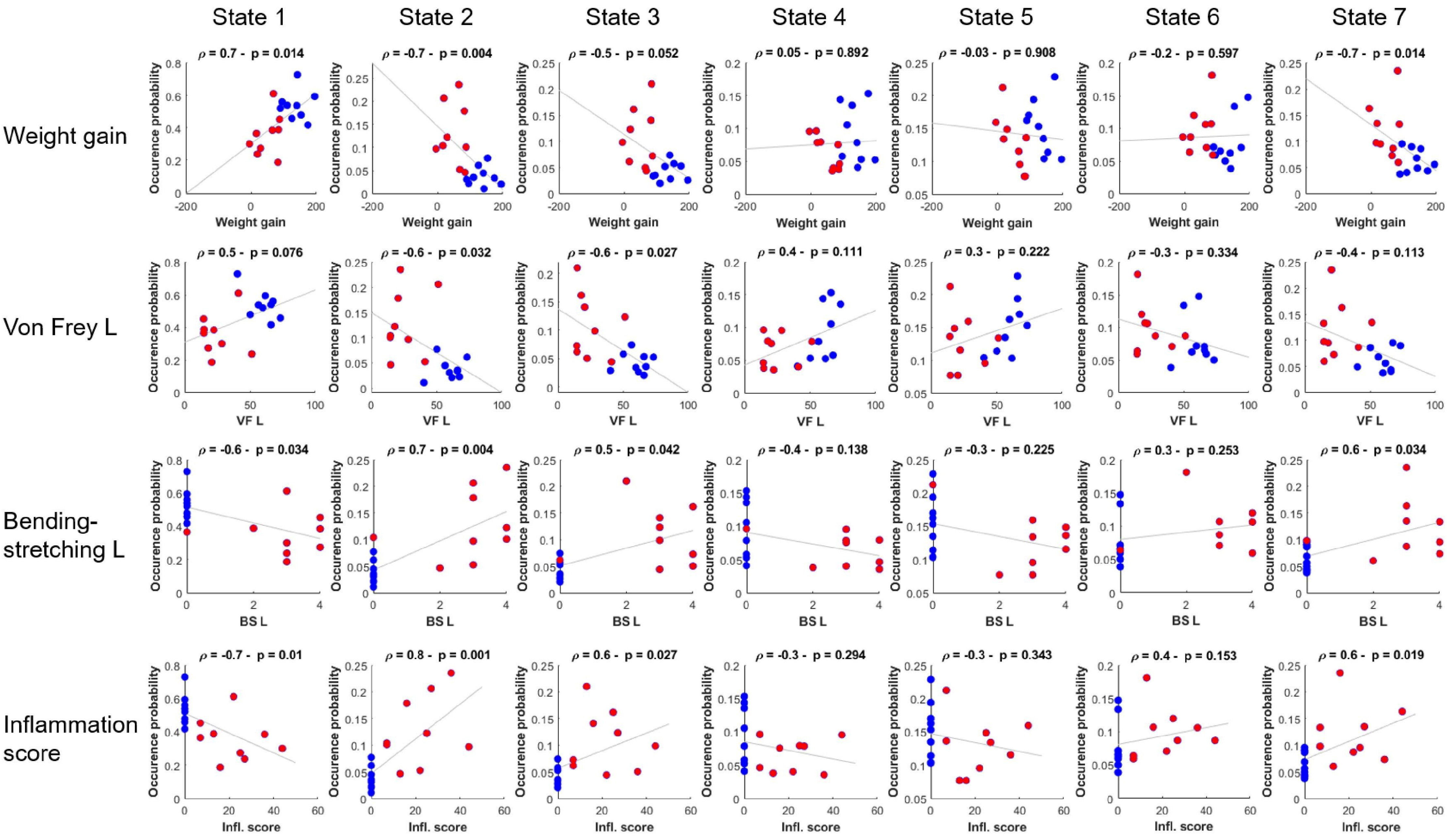

	Name of the plane	Acronyme of the ROI	Full name	Antero posterior location	Lateral location	Location in depth
ROIS in Figure 3	Bregma +4.2 mm	M1-M2 R/L	Right versus left primary and secondary motor cortex	Bregma +4.2 mm	0.5 - 4.9mm	1 - 3mm
		M1-M2	Primary and secondary motor cortex	Bregma +4.2 mm	0.5 - 4.9mm	1 - 3mm
		LO-VO	Lateral and ventral orbital cortex	Bregma +4.2 mm	0.5 - 3.5mm	4 - 6mm
		PrL R/L	Right and left prelimbic cortex	Bregma +4.2 mm	0 - 1mm	3 - 5mm
	Bregma -0.6 mm	Sensorimotor R/L	Right and left sensorimotor cortex (Primary sensory cortex - hind limb part, Primary and secondary motor cortices)	Bregma -0.6 mm	0.5 - 3.8mm	4 - 6mm
		ACC R/L	Right versus left anterior cingulate cortex	Bregma -0.6 mm	0 - 1.5mm	0.5 - 3.5mm
		ACC L	Right and left anterior cingulate cortex	Bregma -0.6 mm	0 - 1.5mm	0.5 - 3.5mm
		ACC R	Right and left anterior cingulate cortex	Bregma -0.6 mm	0 - 1.5mm	0.5 - 3.5mm
		Sensorimotor R	Right sensorimotor cortex (Primary sensory cortex - hind limb part, Primary and secondary motor cortices)	Bregma -0.6 mm	0.5 - 3.8mm	0.5 - 3.5mm
		Sensorimotor L	Left sensorimotor cortex (Primary sensory cortex - hind limb part, Primary and secondary motor cortices)	Bregma -0.6 mm	0.5 - 3.8mm	0.5 - 3.5mm
	Bregma -3.84 mm	Neocortex R/L	Right versus left neocortical region (lateral and medial parietal association cortices)	Bregma -3.84 mm	1.5 - 4.8mm	0.5 - 2.5mm
		RSD	Retrosplenial dysgranular cortex	Bregma -3.84 mm	0.2 - 1.6mm	1 - 3mm
		RSGc R/L	Right versus left retrosplenial granular cortex	Bregma -3.84 mm	0 - 1mm	0.5 - 2mm
		RSGc L	Left retrosplenial granular cortex	Bregma -3.84 mm	0 - 1mm	0.5 - 2mm
		Neocortex L	Left neocortical region (lateral and medial parietal association cortices)	Bregma -3.84 mm	1.5 - 4.8mm	0.5 - 2.5mm
		Hippocampus R/L	Right versus left hippocampus (Dentate gyrus, CA1, CA3, CA2)	Bregma -3.84 mm	0 - 5mm	2.2 - 4.8mm
		Thalamus R/L	Right versus left lateral thalamus (posterior, ventral posteromedial and posterolateral thalamic nuclei)	Bregma -3.84 mm	1.2 - 4mm	5 - 7mm
	Lateral +0.4 mm	Hippocampus	Hippocampus (Dentate gyrus, CA1, CA3, CA2)	Bregma -1.5 / -5 mm	+0.4 mm	3 - 4mm
		Neocortex	Neocortical region (Primary and secondary cingulate cortex, prelimbic cortex)	Bregma +5 / +3.5 mm	+0.4 mm	1 - 4.5mm
		MO	Medial orbital cortex	Bregma +4.5 / -8 mm	+0.4 mm	4.5 - 5.5mm
	Lateral +2.9 mm	Cpu	Caudate Putamen	Bregma +1.5 / -0.5 mm	+2.9 mm	0 - 7mm
		Sensorimotor	Sensorimotor cortex (Primary motor, Primary sensory cortex: forelimb, hind paw and trunck parts, lateral parietal association cortex, primary and secondary visual cortex)	Bregma +3.5 / -9 mm	+2.9 mm	0 - 4mm
		Hippocampus	Hippocampus (Dentate gyrus, CA1, CA3, CA2)	Bregma -2.5 / -6 mm	+2.9 mm	2.1 - 4.1mm
		LO	Lateral orbital cortex	Bregma +2 / +4 mm	+2.9 mm	5 - 6mm
ROIS in Figure 4	Bregma -0.6 mm	Cg1 R	Right primary cingulate cortex	Bregma -0.6 mm	0 - 1.5mm	0.5 - 3.5mm
		Cg1 L	Left primary cingulate cortex	Bregma -0.6 mm	0 - 1.5mm	0.5 - 3.5mm
		Cg2 L	Right secondary cingulate cortex	Bregma -0.6 mm	0 - 1.5mm	0.5 - 3.5mm
		Cg2 R	Left secondary cingulate cortex	Bregma -0.6 mm	0 - 1.5mm	0.5 - 3.5mm
		M1 R	Right primary motor cortex	Bregma -0.6 mm	0 - 2.5mm	0.5 - 3mm
		M1 L	Left primary motor cortex	Bregma -0.6 mm	0 - 2.5mm	0.5 - 3mm
		M2 R	Right secondary motor cortex	Bregma -0.6 mm	0 - 2.5mm	0.5 - 3mm
		M2 L	Left secondary motor cortex	Bregma -0.6 mm	0 - 2.5mm	0.5 - 3mm
		S1HL R	Right somatosensory cortex - Hind limb part	Bregma -0.6 mm	2.4 - 3.8mm	0.5 - 3.5mm
		S1HL L	Left somatosensory cortex - Hind limb part	Bregma -0.6 mm	2.4 - 3.8mm	0.5 - 3.5mm

Supplementary table 1

	ROI couples	Mean correlation controls	Mean correlation arthritics	p-values
Bregma +4.2 mm	M1-M2 R/L	0,86	0,73	*0,21*
	M1-M2 / LO-VO	0,78	0,8	*0,97*
	PrL R/L	0,89	0,75	*0,21*
Bregma -0.6 mm	Sensorimotor R/L	0,79	0,49	***0,002***
	ACC R/L	0,93	0,86	*0,06*
	ACC L / Sensorimotor R	0,66	0,43	***0,02***
	ACC R/ Sensorimotor L	0,64	0,54	*0,19*
	ACC L / Sensorimotor L	0,63	0,53	*0,12*
Bregma -3.84 mm	Neocortex R/L	0,72	0,63	*0,44*
	RSD-RSGc R/L	0,88	0,82	*0,66*
	RSD-RSGc L / Neocortex L	0,62	0.66	*0,71*
	Hippocampus R/L	0,81	0,78	*0,71*
	Thalamus R/L	0,59	0,41	*0,44*
Lateral +0.4 mm	Hippocampus / Neocortex	0,73	0,52	***0,01***
	MO / Neocortex	0,68	0,55	*0,57*
Lateral +2.9 mm	CPu / Sensorimotor	0,3	0,36	*0,73*
	Hippocampus / Sensorimotor	0,53	0.44	*0,46*
	Hippocampus / LO	0,47	0,38	*0,13*
	LO / Sensorimotor	0,52	0,36	***0,02***

Supplementary table 2

ROI couples	Mean correlation controls	Mean correlation arthritics	p-values
S1HL L - M2 L	0,79	0,67	0,05
S1HL L - M2 R	0,70	0,52	**0,04**
S1HL L - M1 R	0,75	0,50	**0,01**
S1HL L - R	0,76	0,59	**0,00**
M1 L - M2 L	0,72	0,54	**0,01**
M1 L - Cg1 L	0,76	0,59	**0,04**
M1 L - M2 R	0,82	0,58	**0,02**
M1 L -M1 R	0,82	0,51	**0,01**
M1 L - S1HL R	0,77	0,44	**0,00**
M2 L - M2 R	0,86	0,67	**0,04**
M2 L - M1 R	0,81	0,56	**0,01**
M2 L - S1HL R	0,75	0,46	**0,01**
Cg1 L - Cg2 L	0,91	0,82	**0,03**
Cg1 L - Cg2 R	0,87	0,76	**0,04**
Cg1 L - Cg1 R	0,91	0,81	0,05
Cg1 L - M2 R	0,84	0,66	**0,03**
Cg1 L - M1 R	0,71	0,52	**0,04**
Cg1 L - S1HL R	0,64	0,42	**0,03**
Cg2 L - Cg2 R	0,93	0,87	**0,04**
Cg2 L - Cg1 R	0,89	0,78	**0,03**
Cg2 L - M2 R	0,77	0,60	**0,03**
Cg2 L - M1 R	0,63	0,46	**0,03**
Cg2 L - S1HL R	0,56	0,38	**0,03**
Cg1 R - Cg2 R	0,90	0,82	**0,04**
Cg2 R - M2 R	0,77	0,62	**0,03**
Cg2 R - M1 R	0,61	0,45	**0,04**
Cg2 R - S1HL R	0,54	0,36	**0,04**
Cg1 R - M2 R	0,88	0,77	**0,04**
Cg1 R - M1 R	0,73	0,59	**0,04**
Cg1 R - S1HL R	0,64	0,46	**0,04**
M1 R - M2 R	0,89	0,81	**0,03**
M2 R - S1HL R	0,81	0,66	**0,04**

Supplementary table 3

		Bregma +4.2 mm				Bregma -0.6 mm				Bregma -3.84 mm				Lateral +0.4 mm				Lateral +2.9 mm			
		Weight index	Von Frey	Bending	Infl.	Weight index	Von Frey	Bending	Infl.	Weight index	Von Frey	Bending	Infl.	Weight index	Von Frey	Bending	Infl.	Weight index	Von Frey	Bending	Infl.
**Seed-based**	SM R/L					+	+		-												
	Cg L - SM R								-												
	Hip - SM						++	-	-												
	LO - SM					+			-												
**Correlation matrix**	S1HL L - M2 L																				
	S1HL L - M2 R								-												
	S1HL L - M1 R								-												
	S1HL L - R					+			-												
	M1 L - M2 L																				
	M1 L - Cg1 L					+			-												
	M1 L - M2 R								-												
	M1 L -M1 R					+	+	--	-												
	M1 L - S1HL R					+	++	--	-												
	M2 L - M2 R								-												
	M2 L - M1 R					+	+	--	-												
	M2 L - S1HL R					+	+	--	-												
	Cg1 L - Cg2 L					+	+	--	-												
	Cg1 L - Cg2 R						++														
	Cg1 L - Cg1 R																				
	Cg1 L - M2 R								-												
	Cg1 L - M1 R								-												
	Cg1 L - S1HL R					+			-												
	Cg2 L - Cg2 R																				
	Cg2 L - Cg1 R						+	-	-												
	Cg2 L - M2 R						+	-	-												
	Cg2 L - M1 R					+	+	--	-												
	Cg2 L - S1HL R								-												
	Cg1 R - Cg2 R					+	++	--	-												
	Cg2 R - M2 R					+		--	-												
	Cg2 R - M1 R								-												
	Cg2 R - S1HL R								-												
	Cg1 R - M2 R					+		--	-												
	Cg1 R - M1 R								-												
	Cg1 R - S1HL R								-												
	M1 R - M2 R								-												
	M2 R - S1HL R								-												
	M2 - PAG														++						
	Cg1 - Hippocampus														+						
	RSD/RSG - Hippocampus														++						
**k-means**	1					+		--	-	-		-						+	++	--	-
	2					-	-	++	+									-			+
	3						-	+	+												
	4																		+		
	5																				
	6																				
	7					-		++	+												

Supplementary table 4

Figure legends of supplementary files

Supplementary Figure 1: Box plots of the occurrence rate of states 4, 5 and 6, depending on the decomposition value k, for the results obtained at Bregma -0.6 mm.

There is no statistical difference between the occurrence probabilities of the states 4, 5 and 6 in the k-means decomposition into 5, 6 and 7 brain states.

Supplementary Figure 2: Dynamic brain state changes in control and arthritic rats imaged in the planes coronal Bregma -3.84 mm (A) and sagittal lateral +2.9 mm (B). Decomposition into brain states 5, 6 and 7 obtained by unsupervised k-means clustering of the phase-locked matrices for the ROIs imaged at Bregma -3.84 mm (A) and Lateral +2.9 mm (B). The p-values written under each matrix show significant differences in the states occurrences between control and arthritic rats. The panels (a) illustrate the Doppler image in the imaging plane, and the overlapping brain atlas (Paxinos and Watson, 1997), showing the ROIs analysed in this plane. (b) are reporting the lists of ROIs, (c) are the matrices for the different states and the p value and (d) are the box plots presenting the occurrence rate of the states statistically significantly modified in this imaging plan. N=9 per group. * $p < 0.05$, ** $p < 0.01$. Delineations of brain areas in A-a and B-b are given with permission from Elsevier. These images were modified from 'Rat Brain in Stereotaxic Coordinates'. Paxinos &Watson, Academic Press, San Diego 3rd, (1997).

Supplementary Figure 3: Scatter plots showing individual correlations between changes in various behaviour items (horizontally: weight gain, mechanical allodynia measured using the electronic Von Frey test on the left hind paw, bending score of the left paw and inflammation score) and occurrence of dynamical FC brain states in control (filled blue circles) and arthritic (filled red circles) rats. Each circle represents an individual animal.

Supplementary Table 1: Table summarizing the list of ROI included in the study, their full name, plane of imaging and coordinates (in antero-posterior, lateral and depth in the rat brain).

Supplementary Table 2: Table summarizing the results obtained using the seed-based analysis in all the imaging planes imaged and for all the ROI pairs tested. Specified are the mean correlation coefficients for both the control group and the arthritic group, and the corresponding p-values (Welch's test if the distribution was Gaussian, Mann-Whitney test otherwise), after a multiple comparison correction (Benjamini-Hochberg correction with a significance threshold of 0.05).

Supplementary Table 3: Table summarizing all the ROI couples tested at Bregma -0.6 mm with the correlation matrices analysis. Specified are the mean correlation coefficients for the control group, the mean correlation coefficients for the arthritic group, and the corresponding p-values (Welch's test if the distribution was Gaussian, Mann-Whitney test otherwise) after multiple comparisons correction (Benjamini-Hochberg correction with a significance threshold of 0.05). The ROIs are the one used and defined in Figure 3.

Supplementary Table 4: Table summarizing the statistically significant correlations (+) or anti-correlations (-) between individual changes in FC analysed using the seed-based analysis, the correlation matrices analysis, or dynamical FC analysis, with the animals' behaviour. Behaviour tests are: weight gain, mechanical allodynia (measured using Von Frey test (VF), bending score (B-S) and inflammation score (Infl.). ++ indicates a positive correlation for the

two paws, while –- indicates an anti-correlation for the two paws.
